# Effect of Dietary *Perilla frutescens* Seed Powder Supplementation on Performance, Egg Quality, and Yolk Fatty Acid Composition of Laying Hens

**DOI:** 10.3390/vetsci13010062

**Published:** 2026-01-09

**Authors:** Yefei Zhou, Zhiding Zhou, Cunyi Qiu, Meilin Yang, Yao Cai, Jun Yuan, Zhihua Feng, Xuezhao Li, Xinglong Wang

**Affiliations:** 1 Department of Food Science, Nanjing Xiaozhuang University, Nanjing 211171, China; 17309367693@163.com (M.Y.);; 2 College of Veterinary Medicine, Sichuan Agricultural University, Chengdu 611130, China; zhidingzhou@163.com; 3 College of Veterinary Science and Technology, Gansu Polytechnic College of Animal Husbandry & Engineering, Wuwei 733006, China; vetqcy5495@nwafu.edu.cn (C.Q.);; 4 College of Veterinary Medicine, Northwest A&F University, Yangling 712100, China; wxlong@nwsuaf.edu.cn

**Keywords:** perilla seed, performance, egg quality, fatty acids, laying hens

## Abstract

In this study, we examined the influence of dietary PFS powder supplementation on production performance, egg quality, and yolk fatty acid profile in laying hens. A total of 192 Hy-Line^®^ Brown hens, 30 weeks of age, were randomly allocated to four dietary treatments containing 0, 30, 60, and 90 g/kg of PFS powder, administered over a 12-week period. No significant differences were observed in egg weight, feed intake, or feed conversion ratio among the treatment groups (*p* > 0.05). However, supplementation with 60 and 90 g/kg PFS significantly enhanced egg production and total egg mass (*p* < 0.05), particularly during weeks 41–44. Egg quality parameters—including albumen height, Haugh unit, yolk color, shell thickness, and shell strength—remained unaffected across treatments (*p* > 0.05). Serum analyses revealed that PFS supplementation significantly reduced levels of total cholesterol, low-density lipoprotein (LDL), triglycerides, and yolk total cholesterol compared with the control diet (*p* < 0.05). Moreover, yolk fatty acid composition was notably altered: total PUFAs and n-3 PUFAs increased (*p* < 0.05), whereas total monounsaturated fatty acids and the n-6/n-3 PUFA ratio decreased (*p* < 0.05) with rising PFS inclusion. In conclusion, dietary PFS powder improved laying performance and favorably modulated yolk fatty acid composition, without compromising egg quality in laying hens.

## Simple Summary

*Perilla frutescens* seed (PFS) is a nutrient-rich resource, containing substantial levels of protein, unsaturated fatty acids, and phenolic compounds, which make it a promising plant-based additive for animal feed. This study evaluated the impact of dietary supplementation with PFS powder on laying performance, egg quality, and yolk fatty acid profile in laying hens. The findings demonstrated that PFS inclusion significantly enhanced laying rate and total egg mass, and increased the concentrations of total polyunsaturated fatty acids (PUFAs) and n-3 PUFAs in egg yolks, without adversely affecting egg quality measures.

## 1. Introduction

While successfully implemented dietary guidelines to reduce total fat intake could lower the consumption of harmful saturated fatty acids (SFAs), they would concurrently diminish the supply of beneficial fatty acids (FAs), such as n-3 fatty acids, whose intake is already considered insufficient [1]. Bioactive compounds of animal origin, especially PUFAs, have garnered growing attention for their beneficial health properties. PUFAs contribute critically to cellular membrane structure and function, support visual and neurological development, and are involved in immune system regulation [2]. Accordingly, significant efforts have been directed toward modifying livestock diets to alter the fatty acid profiles of animal-derived products [3]. In poultry science, substantial work has focused on dietary interventions designed to adjust the fatty acid composition of eggs, even though hens possess endogenous pathways for omega-3 PUFA synthesis [4]. Enriching eggs with omega-3 PUFAs represents a viable strategy to augment the dietary intake of these nutritionally significant lipids, some of which are associated with mitigating the risk of chronic nutrition-related diseases [5,6]. The incorporation of omega-3 PUFAs into yolk lipids can be achieved through dietary supplementation of hens with plant-based sources rich in α-linolenic acid (ALA, C18:3 n-3). ALA serves as the metabolic precursor for the synthesis of longer-chain n-3 derivatives, including eicosapentaenoic acid (EPA), docosapentaenoic acid (DPA), and docosahexaenoic acid (DHA)—compounds widely recognized for their potential health benefits in humans [7,8].

*Perilla frutescens* L. Britton (PF, commonly known as zi-su in Chinese), a member of the Labiatae family, is an edible plant. Its stems, leaves, and seeds find extensive application in traditional Chinese medicine [9]. PF plays an important role and involved in various prescription of Chinese herbals for treating common cold, headache, cough, distention, nausea and bronchial asthma in clinical applications [10]. PFS are characterized by a notable oil content ranging from 35% to 45%, with a high proportion of unsaturated fatty acids, particularly ALA, which constitutes over 60% of the total fatty acids. This composition supports their potential utility in pharmaceutical and food applications. Furthermore, the meal obtained after perilla seed oil extraction is enriched with protein (approximately 40%), phenolic compounds, phytic acid, and polysaccharides [11,12]. Given its nutritional profile, PFS demonstrates potential as a valuable ingredient in livestock feed. Notably, PFS contains high levels of ALA, which are comparable to those found in flaxseed and chia—both recognized for their elevated ALA content—and exceed that of many other plant seed oils [13]. Consequently, incorporating PFS into animal diets could modify the fatty acid composition of derived food products, offering a viable strategy for producing n-3 enriched eggs.

Unlike previous studies concentrating on perilla oil or defatted meal, this study innovatively evaluates whole PFS powder as a multi-nutrient feed ingredient. The specific objectives include: (i) determining the dose-dependent effects of whole PFS powder on laying performance and yolk cholesterol content; and (ii) assessing the feasibility of using whole PFS powder to promote n-3 PUFA deposition for improved economic returns.

## 2. Materials and Methods

### 2.1. Birds and Housing

A total of 192 Hy-Line^®^ Brown laying hens 30 weeks of age were selected for this study, sourced from a commercial hatchery (Nanjing Poultry Institute Hatchery, Nanjing, China). Prior to 30 weeks of age, all hens were maintained under consistent growth and experimental conditions, housed in three-tiered stepped cages equipped with nipple drinkers, with ad libitum access to feed and water. The experiment was conducted at the Nanjing Xiaozhuang University Poultry Farm. Hens were housed in three-tiered stepped cages at a density of three birds per cage, with cage dimensions of 39 cm × 35 cm × 38 cm (length × width × height). The birds were kept in a fully enclosed, mechanically ventilated facility under semi-controlled environmental conditions. Throughout the experimental period, a 16 h light/8 h dark photoperiod was maintained, with ambient temperature controlled at 25 ± 1 °C. Continuous monitoring of behavioral changes in the hens was performed. All experimental protocols were approved by the Animal Care and Ethics Committee of Nanjing Xiaozhuang University (approval number: IACECNXU20170916) and complied with the International Guiding Principles for Biomedical Research Involving Animals.

### 2.2. Seeds Sample and Experimental Diets

PFS powder was purchased from Nanjing Zelang Biological Technology Co., Ltd. (Nanjing, China). The raw PFS material was subjected to dry-heat sterilization at 83 °C, followed by quality assurance testing, and then ground into a powdered form. The powdered material was passed through a sieve to collect particles under 1000 μm. These particles were then vacuum-sealed and kept in darkness at 4 °C until being incorporated into the experimental diets. All compositional analyses of PFS and related laboratory procedures were conducted by a commercial analytical facility (Jiangsu Product Quality Testing & Inspection Institute, Nanjing, China). The major nutrient composition of PFS was analyzed for crude protein, crude fat and dry matter in the laboratory. The AMEn (Nitrogen-Corrected Apparent Metabolizable Energy) content of PFS was assumed at 28.0 MJ/kg based on a previous report [14]. The composition of these ingredients is shown in Table 1.

Prior to the 12-week experimental feeding period (weeks 33–44), hens underwent a two-week acclimatization phase. Following the adaptation period, the hens were assigned randomly four treatments and each treatment included eight replicates of 6 birds.

Hens were fed diets based on corn-soybean meal, and the diets were formulated to meet or exceed nutrient requirements of laying hens based on recommendations [15] and Agricultural Trade Standard of China (NY/T33-2004) [16]. The hens were fed diets in mash form during the experiment. PSF powder was incorporated into the basal diet at graded concentrations of 0 (control), 30, 60, and 90 g/kg. To ensure uniform distribution, the diets with the highest and lowest PSF inclusion levels were initially prepared separately and then blended to obtain the intermediate concentrations. All experimental diets were formulated to be isoenergetic and isonitrogenous, with adjustments made to the proportions of carbohydrate and protein sources. The detailed ingredient list and nutrient composition of the diets are presented in Table 2. The calcium content varied among different diets (3.10–4.76%), which resulted from the substitution of soybean oil with PFS (rich in calcium, as shown in Table 1: 0.26%) and the adjustment of limestone to maintain dietary electrolyte balance. Previous studies have demonstrated that eggshell quality remains stable within the dietary calcium range of 3–5%, thus calcium content was not included as a covariate in the model.

### 2.3. Performance of Laying Hens

Throughout the 12-week experimental period, daily mortality was monitored. Eggs were collected daily, and hen-day egg production percentage was calculated for three intervals: period 1 (weeks 33–36), period 2 (weeks 37–40), and period 3 (weeks 41–44), as well as for the entire study. Daily egg weight (excluding broken eggs) was recorded, and the average egg weight per hen was derived by dividing the total egg weight by the number of laying days. Egg mass production per hen per day was determined by multiplying the laying percentage by the average egg weight. Feed intake per cage was recorded weekly. The weekly feed consumption of each cage was corrected for mortality within that cage to calculate the average feed intake per hen. At the end of the experiment, FCR were calculated as grams of feed consumed per gram of egg mass for each period.

### 2.4. Egg Quality Parameters

Egg quality was assessed at 4-week intervals throughout the trial. From each treatment group, 24 eggs (3 eggs per replicate) were randomly sampled for analysis of albumen height, Haugh unit, yolk color, eggshell thickness, and eggshell strength. Albumen height, Haugh unit, and yolk color were determined using an EggAnalyzer^®^(Robotmation EMT-5200, Tokyo, Japan). Eggshell thickness was measured at three locations (the blunt end, pointed end, and equator) with an Egg Shell Thickness Gauge. Eggshell strength was determined by the Egg Force Reader. All of the equipment was from Orka Food Technology Co., Ltd. (Ramat HaSharon, Israel).

### 2.5. Blood Lipids and Yolk Total Cholesterol Analysis

Following egg quality assessment in each period, yolks were collected, separated from albumen and shell, and stored at −20 °C for subsequent analysis of total cholesterol. Samples of blood (approximately 2 mL) were drawn from the brachial veins of 16 birds (2 birds per replicate) from each treatment at 36, 40 and 44 weeks for the biochemical parameter assay. Serum was separated by centrifuging blood samples at 3000× *g* for 10 min. The resulting supernatant was used to determine lipid profiles, including total cholesterol, triacylglycerols, and low-density lipoprotein cholesterol. The total cholesterol of egg yolk and serum was measured by the enzymatic photometric method (CHOD-PAP method). Serum triacylglycerols were analyzed using the glycerol phosphate oxidase-peroxidase method, while low-density lipoprotein cholesterol (LDL-C) was quantified via a direct assay. Total cholesterol concentrations were measured on a fully automated biochemistry analyzer (WHYA6, Shanghai, China) with commercial reagent kits, following the manufacturer’s instructions (Nanjing Jiancheng Bioengineering Institute, Nanjing, China).

### 2.6. Fatty Acid Profile

At 44 weeks of age, 16 eggs (2 per replicate) were randomly collected from each treatment group for fatty acid analysis. The fatty acid composition of dietary ingredients (Table 3) and egg yolk samples was determined by gas chromatography using a modified procedure based on a previously described method [17]. Briefly, lipids were extracted from samples using a benzene/petroleum ether mixture (2:1, *v*/*v*). The extracted lipids were then combined with 2 mL of toluene and 2 mL of KOH–CH_3_OH (0.4 mg/mL), and incubated at 50 °C for 30 min. Subsequently, 2 mL of 14% BF_3_–CH_3_OH was added, followed by heating at 60 °C for an additional 30 min. Fatty acid methyl esters (FAMEs) were recovered by extraction with 3 mL of 5% NaCl and 1 mL of hexane. FAMEs separation was performed on an Agilent 7890 B gas chromatography system (Agilent Technologies, Santa Clara, CA, USA) equipped with a flame ionization detector and a fused silica capillary column (30 m × 0.32 mm I.D., 0.25 µm film thickness; Supelco Inc., Bellefonte, PA, USA). Nitrogen served as the carrier gas at a flow rate of 0.5 mL/min, with a split ratio of 10:1. The oven temperature was initially held at 100 °C for 5 min, increased to 220 °C at 4 °C/min, and finally maintained at 220 °C for 15 min. Injector and detector temperatures were set at 250 °C and 260 °C, respectively. FAMEs were identified by comparing retention times with those of authentic standards. Quantification was performed using C19:0 methyl ester as an internal standard, which was added prior to extraction. Results are expressed as the relative percentage of each fatty acid to the total fatty acids identified.

### 2.7. Statistical Analysis

Statistical analyses were conducted using the SPSS(version 22.0) software package (SPSS Inc., Chicago, IL, USA). Performance and egg quality data were grouped into four experimental periods: 33–36 weeks, 37–40 weeks, 41–44 weeks, and the entire duration from 33 to 44 weeks of age. For performance parameters, each cage (representing an individual hen) served as the experimental unit in the analysis. A one-way ANOVA was applied to assess treatment effects based on the general linear model: Yij = μ + αi + eij, where Yij represents the observed response, μ the overall mean, αi the fixed effect of PFS powder supplementation, and eij the random residual error. Post hoc comparisons among treatment means were performed using Duncan’s multiple range test, with statistical significance set at *p* < 0.05. Additionally, orthogonal polynomial contrasts were employed to examine linear and quadratic trends across the graded dietary levels of PFS powder (CON, 3% PFS, 6% PFS, and 9% PFS).

## 3. Results

### 3.1. Egg Production

Table 4 summarizes the influence of varying dietary levels of PFS powder on production parameters, including hen-day egg production, egg weight, egg mass, feed intake, and feed conversion ratio (FCR) in laying hens. Dietary PFS supplementation did not significantly alter egg weight, feed intake, or FCR (*p* > 0.05). In contrast, hens receiving diets containing 6% or 9% PFS exhibited significantly higher egg production and egg mass during the periods of 41–44 weeks and over the entire trial (33–44 weeks) compared to the control group (*p* < 0.05). No such improvements were observed during the earlier phases (33–36 and 37–40 weeks).

### 3.2. Egg Quality

Table 5 presents the effects of PFS powder supplementation on egg quality parameters. No significant differences were observed across dietary treatments for albumen height, Haugh unit, yolk color, shell thickness, or shell strength (*p* > 0.05).

### 3.3. Yolk and Serum Cholesterol Contents

Table 6 shows the effect of PFS powder supplementation on serum concentrations of lipids and total cholesterol content of egg yolk. Significantly (*p* < 0.05) lower concentrations of serum total cholesterol and LDL cholesterol were observed in the PFS-supplemented group compared to that of the control group at 40 and 44 weeks of age, but not at 36 weeks of age. Additionally, the PFS-treated group exhibited a linear reduction in serum triglyceride concentration compared with the control group at 44 weeks (*p* < 0.05). The inclusion of PFS powder in the diet resulted in a linear reduction in egg yolk cholesterol content at 40 and 44 weeks of age compared to the soybean-based control diet (*p* < 0.05).

### 3.4. Yolk Fatty Acid Profile

The mean percentages of yolk fatty acids across dietary treatments are summarized in Table 7. Total saturated fatty acids—specifically myristic (C14:0), pentadecanoic (C15:0), palmitic (C16:0), and stearic (C18:0) acids—showed a numerical trend with increasing dietary PFS concentration, although the differences were not statistically significant (*p* > 0.05). However, the concentration of palmitic acid was significantly lower in eggs from hens fed PFS-containing diets compared with the control (*p* < 0.05). In contrast, stearic acid levels were significantly higher in eggs from hens receiving the 9% PFS diet relative to the control and lower PFS inclusion groups (*p* < 0.05).

Compared with the control group, the total MUFA content in egg yolk—comprising myristoleic (C14:1), palmitoleic (C16:1), and oleic (C18:1c) acids—decreased significantly (*p* < 0.05) with increasing dietary PFS inclusion. This reduction was largely attributable to a pronounced decline in oleic acid concentration (*p* < 0.05). While total n-6 fatty acid levels were not significantly altered by PFS supplementation, total PUFA content in yolk increased markedly (*p* < 0.05). Furthermore, eggs from PFS-supplemented hens exhibited significantly higher n-3 fatty acid levels—including ALA, EPA, DPA, and DHA—relative to the control group (*p* < 0.05 or *p* < 0.01). Additionally, the n-6/n-3 PUFA ratio decreased linearly with increasing PFS inclusion (*p* < 0.05).

## 4. Discussion

The objective of this study was to investigate the effects of graded dietary inclusion of PFS powder on laying performance, egg quality, and yolk fatty acid composition. Overall, dietary supplementation with PFS powder did not significantly affect egg weight, feed intake, feed conversion ratio, or egg quality parameters. In contrast, egg production and egg mass were significantly improved when PFS was included at concentrations of 6% or 9%. These results are consistent with previous research by Hammershøj and Steenfeldt [18], in which dietary thyme seed powder was found to improve egg production in laying hens. It is noteworthy that the positive effects of PFS on egg production and egg mass in our study were statistically significant only during weeks 41–44 of age. This period aligns with a known physiological decline in hepatic lipid metabolism in aging hens. We propose that prolonged supplementation with PFS powder may confer cumulative protective benefits: the phenolic constituents present in PFS likely act as free-radical scavengers and enhance the activity of endogenous antioxidant enzymes (e.g., SOD and GSH-Px), thereby alleviating age-related hepatic dysfunction and supporting the synthesis of yolk precursors [19,20,21]. This interpretation is corroborated by earlier reports indicating that the inclusion of other oilseeds, such as hempseed and flaxseed, in layer diets does not compromise egg production, overall performance, or egg quality. The observed benefits may be attributed to the rich profile of specific nutrients in these seeds—including crude protein, fats, minerals, and carbohydrates—which collectively support enhanced reproductive performance and egg output [22,23].

Egg cholesterol content has garnered increasing attention from consumers, healthcare professionals, and researchers alike. This concern stems primarily from epidemiological evidence linking elevated dietary cholesterol intake to increased plasma cholesterol levels and a higher risk of coronary artery disease [24]. In the present study, dietary inclusion of PFS powder at various levels significantly reduced serum total cholesterol, LDL cholesterol in hens, and egg yolk cholesterol, likely attributable to a generalized reduction in lipid mobilization. This may refer to the hypolipidemic effects of dietary PFS, which are fairly consistent in lowering serum triacylglycerols, with variable effects on total and LDL cholesterol concentrations [25]. The mechanism underlying the reduction in egg yolk cholesterol by PFS remains incompletely understood. A potential explanation lies in the bioactive compounds present in PFS, including phytosterols and polyphenols, which may contribute to decreased cholesterol synthesis and enhanced clearance of LDL [26,27]. Cholesterol is primarily biosynthesized in the liver of hens, and incorporated into vitellogenin and LDL particles, which are secreted into the bloodstream from the liver and subsequently taken up by growing oocytes via receptor-mediated endocytosis [28]. Thus, a proposed mechanism for the hypocholesterolemic action of PFS on egg yolk involves the upregulation of LDL receptor expression. Another possible explanation might be that a PFS component has an indirect inhibitory effect exerted at the level of HMG-CoA reductase, a key enzyme in cholesterol biosynthesis [29].Furthermore, recent evidence indicates that red perilla extracts can inhibit LDL oxidation and lipid peroxide formation both in vitro and in human subjects. In murine macrophage models, *Perilla frutescens* extracts reduced oxidized LDL uptake and cellular cholesterol influx, while promoting cholesterol efflux from lipid-laden macrophages [30]. Additionally, perilla leaf extract has been reported to alleviate high-fat diet-induced obesity and dyslipidemia, likely through downregulating adipogenic transcription factors and related target genes [31].

Among plant oils rich in PUFAs, P. frutescens seed oil is distinctive for its high ALA content, an omega-3 fatty acid constituting 54–64% of its total fatty acids. Due to the critical role of fatty acids in human metabolism, characterizing the fatty acid profiles of food products is essential. Typically, conventional table eggs are abundant in n-6 PUFAs while being deficient in n-3 fatty acids. Enriching yolk lipids with n-3 PUFAs can be accomplished through dietary supplementation of hens with n-3-rich feeds [32]. As has been well documented, the inclusion of plant-based sources of n-3 PUFA in laying hen diets leads to increases in the n-3 longer chain PUFA content of egg yolk total lipids. As ALA is the primary plant n-3 fatty acid, it stands to reason that this fatty acid should also reflect the major form of n-3 PUFA in the egg yolk. This has been previously observed for total egg yolk lipids for hens consuming hempseed and flaxseed products [33]. PFS oil serves as a notable source of PUFAs, distinguished by its high ALA content, which constitutes 54–64% of total fatty acids—a proportion considerably greater than that found in most other plant oils. In the current study, dietary supplementation with PFS powder significantly elevated the levels of n-3 PUFAs (including linolenic acid, EPA, and DHA) in laying hens. These findings align with earlier reports demonstrating that perilla seed inclusion similarly enhances n-3 PUFA content (such as ALA and EPA) in lambs [34]. Similarly, Neijat et al. (2016) [35] reported that feeding layers a diet with 25% hempseed increased n-3 PUFA levels and decreased SFA concentration in egg yolks, even after 30 days of room-temperature storage. In addition, the biological efficacy of PFS was further evaluated based on the calculated conversion rate of ALA. Feeding a 9% PFS-supplemented diet resulted in a 15.4-fold increase in yolk EPA + DHA content (from 0.09% to 1.48%), while dietary ALA increased by 20-fold (from 0.24% to 5.16%). This corresponds to an ALA conversion rate as high as 28.7%, which is notably greater than the 19% reported for flaxseed [33]. These findings confirm the high bioavailability of ALA from PFS.

There is competition among the enzymes involved in the elongation and desaturation of omega-3 and omega-6 FAs. Delta-6 desaturase is the critical enzyme in these reactions, for which the greatest affinity appears to be conferred by an increases number of double bonds in the C18 substrate [36]. The enzymatic pathway for the synthesis of arachidonic acid from oleic acid is shared by the n-3 fatty acids. The higher contents of longer chain n-3 fatty acids, such as ALA, eicosapentaenoic and docosahexaenoic acid inhibits the Delta-6 desaturase and thereby reduces conversion of oleic to arachidonic acid [37]. Therefore, according to the results of the present study, dietary PFS powder enriched eggs with n-3, while also reducing the n-6 fatty acid content. Omidi et al. (2015) [32] reported that the optimal ratio of n-6 to n-3 should not exceed 2:1 to 4:1. The current data extends these results by providing evidence that both the 6% and 9% PFS powder supplementation responded in a similar fashion.

At current market prices, PFS (6–9% inclusion) costs 1.08–1.62 ¥/kg dietcomparedtoflaxseed (1.50–2.25 ¥/kg). This 38% cost reduction for PFS, coupled with 5.8–7.7% n-3 PUFA enrichment (Table 7), supports its commercial viability. Although the inclusion of 6–9% PFS powder significantly improves egg production and egg mass in laying hens, as well as increases n-3 PUFA concentration in egg yolk (thereby enhancing egg value), it is noteworthy that PFS powder and egg prices fluctuate with market conditions. Consequently, the economic feasibility of PFS as a feed additive for laying hens may depend on context-specific scenarios.

## 5. Conclusions

In summary, dietary supplementation with 6% or 9% PFS powder improved egg production and egg mass in laying hens. PFS inclusion also significantly reduced yolk cholesterol content without adversely affecting egg quality parameters. As a rich source of ALA, PFS represents a promising feed ingredient for enhancing the n-3 fatty acid profile of eggs, including the long-chain derivatives EPA and DHA, with potential commercial applications in producing n-3-enriched eggs. Furthermore, PFS supplementation elevated total PUFA levels in eggs while lowering the n-6/n-3 fatty acid ratio. Its favorable composition—containing essential nutrients and various bioactive compounds—supports the use of PFS both as a dietary component and a functional additive in layer rations.

## Figures and Tables

**Table 1:** Analysis of the Main Nutritional Components and Fatty Acid Profile of PFS Powder.

Composition	Content (%) ^1^
Dry Matter	93.3
Moisture	6.7
Crude Protein	19.9
Crude Fat	48.9
Ash	3.6
Calcium	0.26
Phosphorous	0.69
Fatty Acid Profile ^1^	
C 16:0 Palmitic	8.32
C 18:0 Stearic	3.17
C 18:1 Oleic	17.82
C 18:2 Linoleic	11.39
C 18:3 Linolenic	57.3
Other Identified Fatty Acids ^2^	2.00
Total Identified Fatty Acids	100.00

^1^ Analyzed by GC-FID; values are expressed as a percentage of total identified fatty acid methyl esters (FAMEs). ^2^ Includes minor fatty acids (each <0.5%) such as C14:0, C16:1, C20:0, etc.

**Table 2:** Ingredients and nutrient composition of experimental diets for laying hens.

Items	PFS Powder Supplemental Concentrations (g/kg)			
	0 (Control)	30	60	90
Ingredients (%)				
Corn grain	54.9	53.5	53	47.3
Soybean meal (44% crude protein)	27.4	26.2	24.9	24.5
PFS powder	0	3	6	9
Soybean oil	3.1	1.7	0	0
Dicalcium phosphate	1.7	1.7	1.7	1.7
Limestone	7.4	8.4	8.9	12
Salt	0.3	0.3	0.3	0.3
L-Lisine-HCl (78%)	0.1	0.1	0.1	0.1
*DL*-Methionine (98%)	0.1	0.1	0.1	0.1
Vitamin- mineral premix ^1^	5.0	5.0	5.0	5.0
Total	100.0	100.0	100.0	100.0
Nutrient concentrations ^2^				
ME (MJ/kg)	11.47	11.48	11.50	11.50
Crude protein (%)	16.17	16.14	16.13	16.12
Calcium (%)	3.10	3.46	3.65	4.76
Total phosphorus (%)	0.63	0.64	0.65	0.66
Available phosphorus (%)	0.44	0.45	0.47	0.48
Methionine (%)	0.35	0.34	0.33	0.32
Lysine (%)	0.86	0.91	0.87	0.85
Methionine + Cysteine (%)	0.61	0.59	0.58	0.55

^1^ Provided the following per kilogram of diet: vitamin A, 10,400 IU; vitamin D_3_ 2400 IU; vitamin E, 150 IU; vitamin K_3_, 5.6 mg; vitamin B_1_, 1.4 mg; vitamin B_2_, 6.4 mg; vitamin B_6_, 3.2 mg; vitamin B_12_, 0.024 mg; nicotinic acid, 30 mg; *D*-pantothenic acid, 16.4 mg; folic acid, 0.52 mg; choline chloride, 600 mg; biotin, 0.1 mg; Fe, 120 mg; Cu, 20 mg; Mn, 52 mg; Zn, 120 mg; I, 1.00 mg; Se, 0.32 mg; Co, 1.04 mg. ^2^ The value of crude protein was analyzed and others are calculated values.

**Table 3:** Fatty acid profile of experimental diets.

Fatty Acid (% of Diet)	PFS Powder Supplemental Concentrations (g/kg)			
	0 (Control)	30	60	90
Myristic (C14:0)	0.03	0.04	0.04	0.04
Pentadecanoic (C15:0)	0.00	0.00	0.00	0.00
Palmitic (C16:0)	0.33	0.43	0.51	0.76
Stearic (C18:0)	0.17	0.18	0.19	0.29
Myristoleic (C14:1)	0.00	0.01	0.01	0.02
Palmitoleic (C16:1)	0.01	0.01	0.01	0.01
Oleic (C18:1c)	0.78	0.93	1.07	1.66
linoleic (C18:2 n-6)	1.56	1.19	0.68	1.07
γ-Linolenic (C18:3 n-6)	0.00	0.01	0.02	0.05
Arachidonic (C20:4 n-6)	0.00	0.00	0.00	0.00
α-linolenic (C18:3 n-3)	0.24	1.84	3.44	5.16
Eicosapentaenoic (C20:5 n-3)	0.00	0.00	0.00	0.00
Docosapentaenoic (22:5n-3)	0.00	0.00	0.00	0.00
Docosahexaenoic (C22:6 n-3)	0.00	0.00	0.00	0.00
Total SFA	0.53	0.65	0.74	1.09
Total MUFA	0.79	0.95	1.09	1.69
Total PUFAs	1.80	3.04	4.14	6.28

SFA = saturated fatty acid; MUFA = monounsaturated fatty acid; PUFAs = polyunsaturated fatty acids. Total SFA = sum of C14:0, C15:0, C16:0 and C18:0; Total MUFA = sum of C14:1, C16:1 and C18:1c; Total PUFA = sum of C18:2 n-6, C18:3 n-6, C20:4 n-6, C18:3 n-3, C20:5 n-3, 22:5n-3 and C22:6 n-3.

**Table 4:** The effect of dietary PFS powder on performance of laying hens during 12 weeks.

Parameters	PFS Powder Supplemental Concentrations (g/kg)				SEM	*p*-Value		
	0 (Control)	30	60	90		ANOVA	L	Q
Sample Size	48	48	48	48				
Egg production (%)								
33–36 wk	94.8	94.4	94.2	94.3	1.32	Ns	Ns	Ns
37–40 wk	93.6	94.2	94.7	94.9	0.68	Ns	Ns	Ns
41–44 wk	91.2 ^b^	92.0 ^ab^	93.8 ^a^	94.2 ^a^	1.86	*	*	Ns
33–44 wk	93.2 ^b^	93.5 ^ab^	94.2 ^a^	94.5 ^a^	1.12	*	Ns	Ns
Egg weight (g/egg)								
33–36 wk	61.5	61.7	60.9	61.1	0.24	Ns	Ns	Ns
37–40 wk	59.9	60.1	59.4	60.7	0.17	Ns	Ns	Ns
41–44 wk	60.2	59.5	60.4	60.3	0.27	Ns	Ns	Ns
33–44 wk	60.2	60.4	60.2	60.7	0.03	Ns	Ns	Ns
Egg mass (g/day per hen)								
33–36 wk	58.3	58.2	57.4	57.6	0.18	Ns	Ns	Ns
37–40 wk	56.1	56.6	56.3	57.6	0.27	Ns	Ns	Ns
41–44 wk	54.9 ^b^	54.7 ^b^	56.7 ^a^	56.8 ^a^	0.16	*	Ns	Ns
33–44 wk	56.4 ^b^	56.5 ^b^	56.8 ^b^	57.3 ^a^	0.11	*	Ns	Ns
Feed intake (g/day per hen)								
33–36 wk	108.5	111.8	113.5	119.7	0.57	Ns	Ns	Ns
37–40 wk	110.0	112.1	119.6	119.2	0.19	Ns	Ns	Ns
41–44 wk	109.1	110.3	112.1	112.9	0.69	Ns	Ns	Ns
33–44 wk	109.2	111.4	115.1	117.3	0.61	Ns	Ns	Ns
Feed conversion ratio								
33–36 wk	1.86	1.92	1.98	2.08	0.07	Ns	Ns	Ns
37–40 wk	1.96	1.98	2.12	2.07	0.09	Ns	Ns	Ns
41–44 wk	1.99	2.02	1.98	1.99	0.07	Ns	Ns	Ns
33–44 wk	1.94	1.97	2.03	2.05	0.11	Ns	Ns	Ns

L = linear, Q = quadratic, Ns = non-significant. Means were obtained from eight replicate cages of 6 birds each. ^a,b^ Values in the same row with no common superscripts are significantly different (*p* < 0.05). *, *p* < 0.05.

**Table 5:** Effects of supplementation PFS powder on egg quality parameter of laying hens.

Parameters	PFS Powder Supplemental Concentrations (g/kg)				SEM	*p*-Value		
	0 (Contro)	30	60	90		ANOVA	L	Q
Sample Size	24	24	24	24				
Albumen height (mm)								
36 wk	6.29	6.19	6.27	6.47	0.189	Ns	Ns	Ns
40 wk	6.13	6.19	6.11	5.96	0.247	Ns	Ns	Ns
44 wk	6.26	6.84	6.27	5.87	0.278	Ns	Ns	Ns
Haugh unit								
36 wk	83.55	82.6	83.45	83.9	1.303	Ns	Ns	Ns
40 wk	82.26	82.26	82.24	82.4	1.714	Ns	Ns	Ns
44 wk	84.14	80.31	82.94	82.3	1.994	Ns	Ns	Ns
Egg yolk color								
36 wk	5.89	5.94	5.93	5.89	0.051	Ns	Ns	Ns
40 wk	5.84	5.87	5.86	5.89	0.108	Ns	Ns	Ns
44 wk	5.87	5.96	5.94	5.90	0.102	Ns	Ns	Ns
Eggshell strength, kg/cm^2^								
36 wk	4.35	4.56	4.51	4.57	0.004	Ns	Ns	Ns
40 wk	4.38	4.47	4.53	4.87	0.095	Ns	Ns	Ns
44 wk	4.41	4.42	4.55	4.60	0.059	Ns	Ns	Ns
Eggshell thickness, mm								
36 wk	0.22	0.24	0.22	0.25	0.009	Ns	Ns	Ns
40 wk	0.23	0.26	0.28	0.26	0.009	Ns	Ns	Ns
44 wk	0.22	0.24	0.28	0.24	0.009	Ns	Ns	Ns

Means were calculated on *n* = 8 replicates (3 eggs per replicate) per treatment.

**Table 6:** The effect of supplementation PFS powder on contents of serum lipids and egg yolk in laying hens from 32 to 44 weeks of age.

Parameters	PFS Powder Supplemental Concentrations (g/kg)				SEM	*p*-Value		
	0 (Contro)	30	60	90		ANOVA	L	Q
Sample Size	16	16	16	16				
Serum total cholesterol (mmol/L) ^1^								
36 wk	7.1	6.9	6.7	6.8	0.011	Ns	Ns	Ns
40 wk	7.3 ^c^	5.9 ^b^	5.5 ^b^	4.2 ^a^	0.017	*	*	Ns
44 wk	7.2 ^c^	5.8 ^b^	4.3 ^a^	4.0 ^a^	0.009	*	*	Ns
Serum triacylglycerols (mmol/L) ^1^								
36 wk	1.29	1.25	1.27	1.16	0.003	Ns	Ns	Ns
40 wk	1.27	1.20	1.17	1.09	0.018	Ns	Ns	Ns
44 wk	1.16 ^c^	0.91 ^b^	0.86 ^b^	0.54 ^a^	0.042	*	*	Ns
Serum LDL (mmol/L) ^1^								
36 wk	1.93	1.91	1.94	1.85	0.003	Ns	Ns	Ns
40 wk	1.95 ^c^	1.61 ^b^	1.44 ^a^	1.35 ^a^	0.025	*	*	Ns
44 wk	1.93 ^c^	1.57 ^b^	1.01 ^a^	0.90 ^a^	0.048	*	*	Ns
Egg yolk total cholesterol concentration (mg/g yolk) ^2^								
36 wk	12.88	12.37	12.46	11.59	0.015	Ns	Ns	Ns
40 wk	12.91 ^c^	11.05 ^b^	11.12 ^b^	10.28 ^a^	0.012	*	*	Ns
44 wk	12.83 ^c^	11.15 ^b^	10.24 ^b^	9.81 ^a^	0.018	*	*	Ns

L = linear, Q = quadratic, Ns = non-significant. ^1^ Data are means of 8 replications with 2 hens per replicate. ^2^ Data are means of 8 replications with 3 eggs per replicate. ^a–c^ Means sharing different letters in the same row are significantly different (*p* < 0.05). *, *p* < 0.05.

**Table 7:** Effects of supplementation PFS powder on fatty acid composition of egg yolk.

Fatty Acids Profile (%)	PFS Powder Supplemental Concentrations (g/kg)				SEM	*p*-Value		
	0 (Contro)	30	60	90		ANOVA	L	Q
Sample Size	16	16	16	16				
Myristic (C14:0)	0.45	0.40	0.39	0.43	0.002	Ns	Ns	Ns
Pentadecanoic (C15:0)	0.06	0.07	0.07	0.06	0.003	Ns	Ns	Ns
Palmitic (C16:0)	20.33 ^c^	18.07 ^ab^	17.69 ^b^	15.78 ^a^	0.217	*	*	Ns
Stearic (C18:0)	8.14 ^c^	8.75 ^c^	9.92 ^b^	12.83 ^a^	0.009	*	Ns	Ns
Total SFA	28.98	27.29	28.07	29.10	0.371	Ns	Ns	Ns
Myristoleic (C14:1)	0.10	0.08	0.07	0.07	0.004	Ns	Ns	Ns
Palmitoleic (C16:1)	3.97	3.83	3.54	3.51	0.001	Ns	Ns	Ns
Oleic (C18:1c)	45.30 ^d^	43.75 ^c^	42.21 ^b^	40.10 ^a^	0.121	*	*	*
Total MUFA	48.37 ^d^	47.66 ^c^	45.82 ^b^	43.68 ^a^	0.013	*	*	Ns
LA (C18:2 n-6)	19.76	18.97	18.48	17.29	0.032	Ns	Ns	Ns
GLA (C18:3 n-6)	0.18	0.21	0.26	0.34	0.011	Ns	Ns	Ns
ARA (C20:4 n-6)	1.96	1.82	1.85	1.89	0.006	Ns	Ns	Ns
ALA (C18:3 n-3)	0.57 ^d^	2.60 ^c^	3.93 ^b^	5.79 ^a^	0.008	**	**	*
EPA (C20:5 n-3)	0.03 ^c^	0.11 ^b^	0.14 ^b^	0.21 ^a^	0.012	*	*	Ns
DPA(22:5n-3)	0.06 ^c^	0.17 ^b^	0.19 ^b^	0.22 ^a^	0.004	*	*	Ns
DHA (C22:6 n-3)	0.09 ^c^	1.17 ^b^	1.26 ^b^	1.48 ^a^	0.011	*	*	Ns
Total PUFA	22.65 ^d^	25.05 ^c^	26.11 ^b^	27.22 ^a^	0.046	*	*	*
Total n-6 PUFA	21.90	21.00	20.59	19.52	0.631	Ns	Ns	Ns
Total n-3 PUFA	0.75 ^d^	4.05 ^c^	5.52 ^b^	7.70 ^a^	0.021	*	*	Ns
n-6/n-3 PUFA ratio	29.20 ^d^	5.19 ^c^	3.73 ^b^	2.54 ^a^	6.346	*	*	Ns

L = linear, Q = quadratic, Ns = non-significant, LA = linoleic acid; GLA = γ-Linolenic acid; ARA = arachidonic acid; ALA = α-Linolenic acid; EPA = Eicosapentenoic acid; DHA = Docosahexaenoic acid; SFA = saturated fatty acid; MUFA = monounsaturated fatty acid; PUFA = polyunsaturated fatty acid; Total SFA = sum of C14:0, C15:0, C16:0 and C18:0; Total MUFA = sum of C14:1, C16:1 and C18:1c; Total PUFA = sum of C18:2 n-6, C18:3 n-6, C20:4 n-6, C18:3 n-3, C20:5 n-3, 22:5n-3 and C22:6 n-3; Total n-6 = sum of LA, GLA and ARA; Total n-3 = sum of ALA, EPA, DPA and DHA. Means represent 16 eggs per treatment of 2 eggs per replicate. ^a–d^ Means sharing different letters in the same row are significantly different (*p* < 0.05). *, *p* < 0.05; **, *p* < 0.01.

## Data Availability

The original contributions presented in this study are included in the article. Further inquiries can be directed to the corresponding author.

## Abbreviations

The following abbreviations are used in this manuscript:

PUFA	polyunsaturated fatty acid
PFS	*Perilla frutescens* seed
EPA	eicosapentaenoic acid
DPA	docosapentaenoic acid
DHA	docosahexaenoic acid
FA	fatty acids
SFA	saturated fatty acid
AMEn	Nitrogen-Corrected Apparent Metabolizable Energy
LDL-C	low density lipoprotein cholesterol
